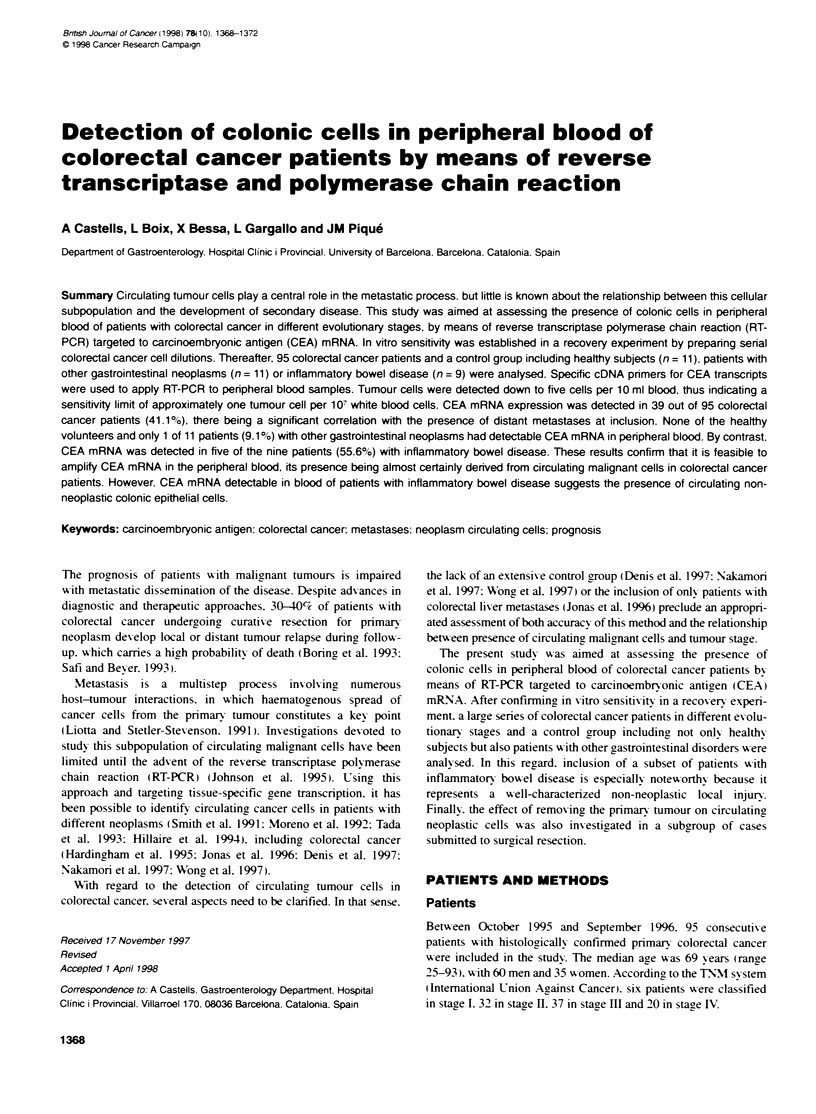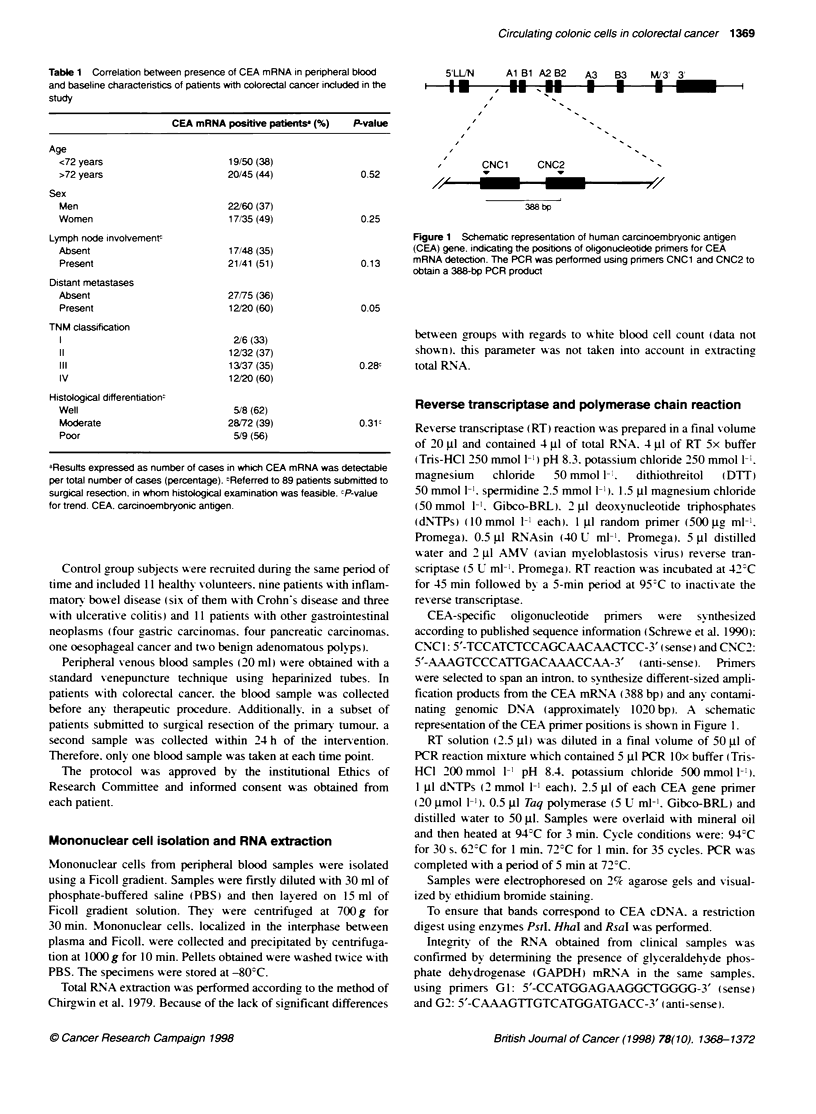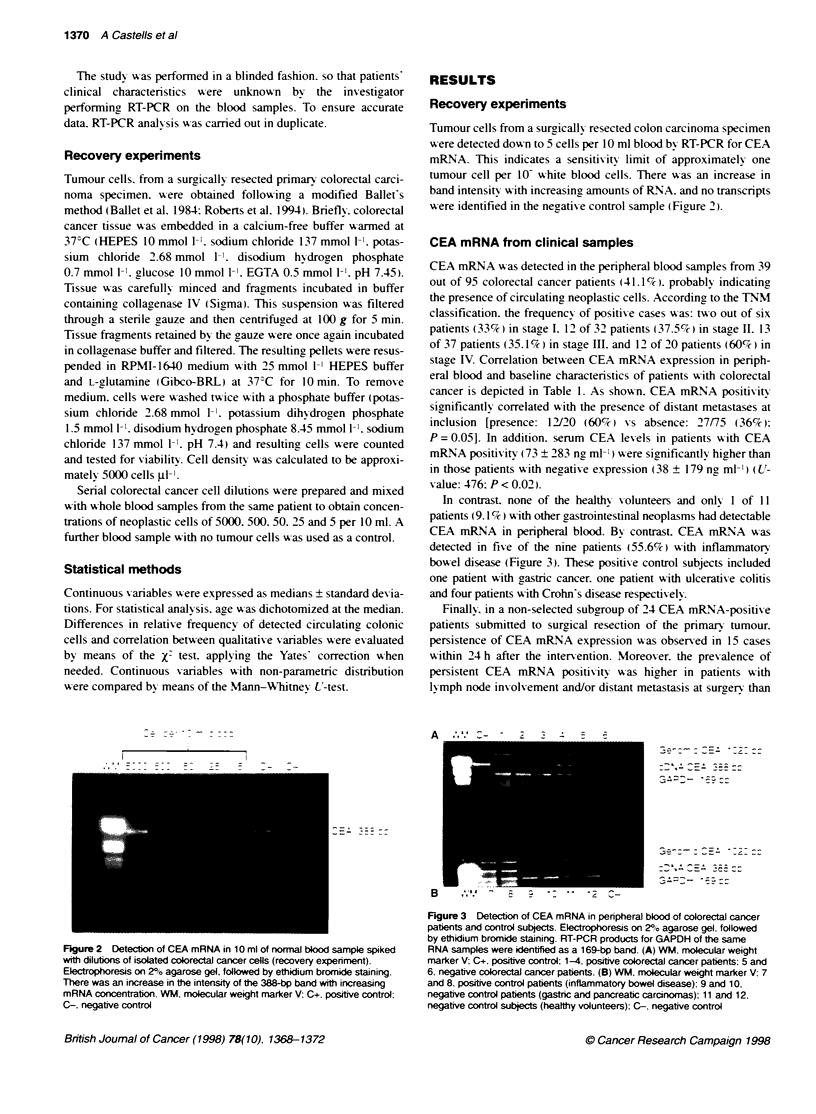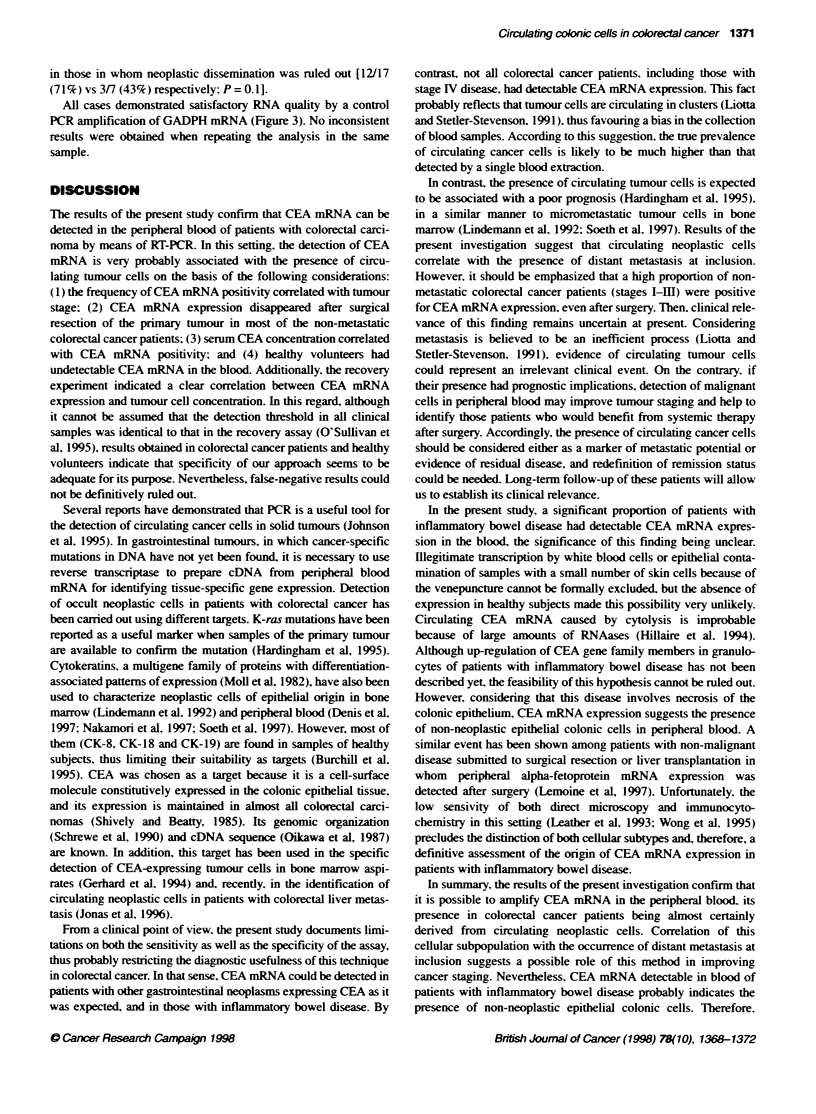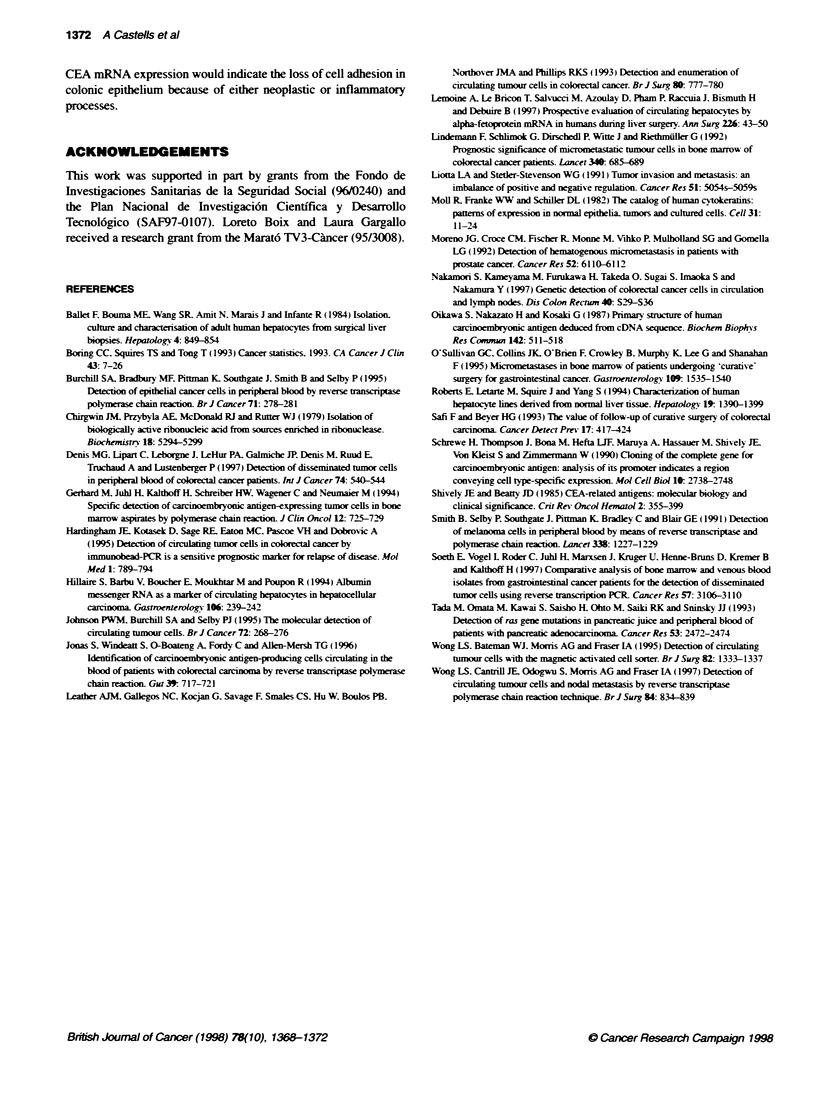# Detection of colonic cells in peripheral blood of colorectal cancer patients by means of reverse transcriptase and polymerase chain reaction.

**DOI:** 10.1038/bjc.1998.686

**Published:** 1998-11

**Authors:** A. Castells, L. Boix, X. Bessa, L. Gargallo, J. M. Piqué

**Affiliations:** Department of Gastroenterology, Hospital Clínic i Provincial, University of Barcelona, Catalonia, Spain.

## Abstract

**Images:**


British Journal of Cancer (1998) 78(10). 1368–1372
© 1998 Cancer Research Campaign

### Detection of colonic cells in peripheral blood of colorectal cancer patients by means of reverse transcriptase and polymerase chain reaction

**A Castells, L Boix, X Bessa, L Gargallo and JM Piqué**

Department of Gastroenterology. Hospital Clinic i Provincial. University of Barcelona. Barcelona. Catalonia. Spain

**Summary** Circulating tumour cells play a central role in the metastatic process. but little is known about the relationship between this cellular subpopulation and the development of secondary disease. This study was aimed at assessing the presence of colonic cells in peripheral blood of patients with colorectal cancer in different evolutionary stages, by means of reverse transcriptase polymerase chain reaction (RT-PCR) targeted to carcinoembryonic antigen (CEA) mRNA. In vitro sensitivity was established in a recovery experiment by preparing serial colorectal cancer cell dilutions. Thereafter, 95 colorectal cancer patients and a control group including healthy subjects ($n$ = 11), patients with other gastrointestinal neoplasms ($n$ = 11) or inflammatory bowel disease ($n$ = 9) were analysed. Specific cDNA primers for CEA transcripts were used to apply RT-PCR to peripheral blood samples. Tumour cells were detected down to five cells per 10 ml blood, thus indicating a sensitivity limit of approximately one tumour cell per $10^7$ white blood cells. CEA mRNA expression was detected in 39 out of 95 colorectal cancer patients (41.1%), there being a significant correlation with the presence of distant metastases at inclusion. None of the healthy volunteers and only 1 of 11 patients (9.1%) with other gastrointestinal neoplasms had detectable CEA mRNA in peripheral blood. By contrast. CEA mRNA was detected in five of the nine patients (55.6%) with inflammatory bowel disease. These results confirm that it is feasible to amplify CEA mRNA in the peripheral blood, its presence being almost certainly derived from circulating malignant cells in colorectal cancer patients. However, CEA mRNA detectable in blood of patients with inflammatory bowel disease suggests the presence of circulating non-neoplastic colonic epithelial cells.

**Keywords:** carcinoembryonic antigen; colorectal cancer; metastases; neoplasm circulating cells; prognosis

The prognosis of patients with malignant tumours is impaired with metastatic dissemination of the disease. Despite advances in diagnostic and therapeutic approaches. 30–40% of patients with colorectal cancer undergoing curative resection for primary neoplasm develop local or distant tumour relapse during follow-up, which carries a high probability of death (Boring et al. 1993; Safi and Beyer. 1993).

Metastasis is a multistep process involving numerous host–tumour interactions, in which haematogenous spread of cancer cells from the primary tumour constitutes a key point (Liotta and Stetler-Stevenson. 1991). Investigations devoted to study this subpopulation of circulating malignant cells have been limited until the advent of the reverse transcriptase polymerase chain reaction (RT-PCR) (Johnson et al. 1995). Using this approach and targeting tissue-specific gene transcription, it has been possible to identify circulating cancer cells in patients with different neoplasms (Smith et al. 1991; Moreno et al. 1992; Tada et al. 1993; Hillaire et al. 1994), including colorectal cancer (Hardingham et al. 1995; Jonas et al. 1996; Denis et al. 1997; Nakamori et al. 1997; Wong et al. 1997).

With regard to the detection of circulating tumour cells in colorectal cancer, several aspects need to be clarified. In that sense, the lack of an extensive control group (Denis et al. 1997; Nakamori et al. 1997; Wong et al. 1997) or the inclusion of only patients with colorectal liver metastases (Jonas et al. 1996) preclude an appropriated assessment of both accuracy of this method and the relationship between presence of circulating malignant cells and tumour stage.

The present study was aimed at assessing the presence of colonic cells in peripheral blood of colorectal cancer patients by means of RT-PCR targeted to carcinoembryonic antigen (CEA) mRNA. After confirming in vitro sensitivity in a recovery experiment, a large series of colorectal cancer patients in different evolutionary stages and a control group including not only healthy subjects but also patients with other gastrointestinal disorders were analysed. In this regard, inclusion of a subset of patients with inflammatory bowel disease is especially noteworthy because it represents a well-characterized non-neoplastic local injury. Finally, the effect of removing the primary tumour on circulating neoplastic cells was also investigated in a subgroup of cases submitted to surgical resection.

*Received 17 November 1997*
*Revised*
*Accepted 1 April 1998*

*Correspondence to:* A Castells. Gastroenterology Department. Hospital Clinic i Provincial. Villarroel 170. 08036 Barcelona. Catalonia. Spain

### PATIENTS AND METHODS

### Patients

Between October 1995 and September 1996, 95 consecutive patients with histologically confirmed primary colorectal cancer were included in the study. The median age was 69 years (range 25–93), with 60 men and 35 women. According to the TNM system (International Union Against Cancer), six patients were classified in stage I, 32 in stage II, 37 in stage III and 20 in stage IV.

**Table 1** Correlation between presence of CEA mRNA in peripheral blood and baseline characteristics of patients with colorectal cancer included in the study

| | CEA mRNA positive patients[a] (%) | P-value |
|---|---|---|
| Age | | |
| <72 years | 19/50 (38) | |
| >72 years | 20/45 (44) | 0.52 |
| Sex | | |
| Men | 22/60 (37) | |
| Women | 17/35 (49) | 0.25 |
| Lymph node involvement[b] | | |
| Absent | 17/48 (35) | |
| Present | 21/41 (51) | 0.13 |
| Distant metastases | | |
| Absent | 27/75 (36) | |
| Present | 12/20 (60) | 0.05 |
| TNM classification | | |
| I | 2/6 (33) | |
| II | 12/32 (37) | |
| III | 13/37 (35) | 0.28[c] |
| IV | 12/20 (60) | |
| Histological differentiation[b] | | |
| Well | 5/8 (62) | |
| Moderate | 28/72 (39) | 0.31[c] |
| Poor | 5/9 (56) | |

[a]Results expressed as number of cases in which CEA mRNA was detectable per total number of cases (percentage). [b]Referred to 89 patients submitted to surgical resection, in whom histological examination was feasible. [c]P-value for trend. CEA, carcinoembryonic antigen.

Control group subjects were recruited during the same period of time and included 11 healthy volunteers, nine patients with inflammatory bowel disease (six of them with Crohn's disease and three with ulcerative colitis) and 11 patients with other gastrointestinal neoplasms (four gastric carcinomas, four pancreatic carcinomas, one oesophageal cancer and two benign adenomatous polyps).

Peripheral venous blood samples (20 ml) were obtained with a standard venepuncture technique using heparinized tubes. In patients with colorectal cancer, the blood sample was collected before any therapeutic procedure. Additionally, in a subset of patients submitted to surgical resection of the primary tumour, a second sample was collected within 24 h of the intervention. Therefore, only one blood sample was taken at each time point.

The protocol was approved by the institutional Ethics of Research Committee and informed consent was obtained from each patient.

### Mononuclear cell isolation and RNA extraction

Mononuclear cells from peripheral blood samples were isolated using a Ficoll gradient. Samples were firstly diluted with 30 ml of phosphate-buffered saline (PBS) and then layered on 15 ml of Ficoll gradient solution. They were centrifuged at 700 *g* for 30 min. Mononuclear cells, localized in the interphase between plasma and Ficoll, were collected and precipitated by centrifugation at 1000 *g* for 10 min. Pellets obtained were washed twice with PBS. The specimens were stored at –80°C.

Total RNA extraction was performed according to the method of Chirgwin et al, 1979. Because of the lack of significant differences between groups with regards to white blood cell count (data not shown), this parameter was not taken into account in extracting total RNA.

**Figure 1** Schematic representation of human carcinoembryonic antigen (CEA) gene, indicating the positions of oligonucleotide primers for CEA mRNA detection. The PCR was performed using primers CNC1 and CNC2 to obtain a 388-bp PCR product

### Reverse transcriptase and polymerase chain reaction

Reverse transcriptase (RT) reaction was prepared in a final volume of 20 µl and contained 4 µl of total RNA, 4 µl of RT 5× buffer (Tris-HCl 250 mmol $l^{-1}$) pH 8.3, potassium chloride 250 mmol $l^{-1}$, magnesium chloride 50 mmol $l^{-1}$, dithiothreitol (DTT) 50 mmol $l^{-1}$, spermidine 2.5 mmol $l^{-1}$), 1.5 µl magnesium chloride (50 mmol $l^{-1}$, Gibco-BRL), 2 µl deoxynucleotide triphosphates (dNTPs) (10 mmol $l^{-1}$ each), 1 µl random primer (500 µg $ml^{-1}$, Promega), 0.5 µl RNAsin (40 U $ml^{-1}$, Promega), 5 µl distilled water and 2 µl AMV (avian myeloblastosis virus) reverse transcriptase (5 U $ml^{-1}$, Promega). RT reaction was incubated at 42°C for 45 min followed by a 5-min period at 95°C to inactivate the reverse transcriptase.

CEA-specific oligonucleotide primers were synthesized according to published sequence information (Schrewe et al, 1990): CNC1: 5′-TCCATCTCCAGCAACAACTCC-3′ (sense) and CNC2: 5′-AAAGTCCCATTGACAAACCAA-3′ (anti-sense). Primers were selected to span an intron, to synthesize different-sized amplification products from the CEA mRNA (388 bp) and any contaminating genomic DNA (approximately 1020 bp). A schematic representation of the CEA primer positions is shown in Figure 1.

RT solution (2.5 µl) was diluted in a final volume of 50 µl of PCR reaction mixture which contained 5 µl PCR 10× buffer (Tris-HCl 200 mmol $l^{-1}$ pH 8.4, potassium chloride 500 mmol $l^{-1}$), 1 µl dNTPs (2 mmol $l^{-1}$ each), 2.5 µl of each CEA gene primer (20 µmol $l^{-1}$), 0.5 µl *Taq* polymerase (5 U $ml^{-1}$, Gibco-BRL) and distilled water to 50 µl. Samples were overlaid with mineral oil and then heated at 94°C for 3 min. Cycle conditions were: 94°C for 30 s, 62°C for 1 min, 72°C for 1 min, for 35 cycles. PCR was completed with a period of 5 min at 72°C.

Samples were electrophoresed on 2% agarose gels and visualized by ethidium bromide staining.

To ensure that bands correspond to CEA cDNA, a restriction digest using enzymes *Pst*I, *Hha*I and *Rsa*I was performed.

Integrity of the RNA obtained from clinical samples was confirmed by determining the presence of glyceraldehyde phosphate dehydrogenase (GAPDH) mRNA in the same samples, using primers G1: 5′-CCATGGAGAAGGCTGGGG-3′ (sense) and G2: 5′-CAAAGTTGTCATGGATGACC-3′ (anti-sense).

© Cancer Research Campaign 1998

The study was performed in a blinded fashion, so that patients' clinical characteristics were unknown by the investigator performing RT-PCR on the blood samples. To ensure accurate data, RT-PCR analysis was carried out in duplicate.

### Recovery experiments

Tumour cells, from a surgically resected primary colorectal carcinoma specimen, were obtained following a modified Ballet's method (Ballet et al, 1984; Roberts et al, 1994). Briefly, colorectal cancer tissue was embedded in a calcium-free buffer warmed at 37°C (HEPES 10 mmol $l^{-1}$, sodium chloride 137 mmol $l^{-1}$, potassium chloride 2.68 mmol $l^{-1}$, disodium hydrogen phosphate 0.7 mmol $l^{-1}$, glucose 10 mmol $l^{-1}$, EGTA 0.5 mmol $l^{-1}$, pH 7.45). Tissue was carefully minced and fragments incubated in buffer containing collagenase IV (Sigma). This suspension was filtered through a sterile gauze and then centrifuged at 100 ***g*** for 5 min. Tissue fragments retained by the gauze were once again incubated in collagenase buffer and filtered. The resulting pellets were resuspended in RPMI-1640 medium with 25 mmol $l^{-1}$ HEPES buffer and L-glutamine (Gibco-BRL) at 37°C for 10 min. To remove medium, cells were washed twice with a phosphate buffer (potassium chloride 2.68 mmol $l^{-1}$, potassium dihydrogen phosphate 1.5 mmol $l^{-1}$, disodium hydrogen phosphate 8.45 mmol $l^{-1}$, sodium chloride 137 mmol $l^{-1}$, pH 7.4) and resulting cells were counted and tested for viability. Cell density was calculated to be approximately 5000 cells $\mu l^{-1}$.

Serial colorectal cancer cell dilutions were prepared and mixed with whole blood samples from the same patient to obtain concentrations of neoplastic cells of 5000, 500, 50, 25 and 5 per 10 ml. A further blood sample with no tumour cells was used as a control.

### Statistical methods

Continuous variables were expressed as medians ± standard deviations. For statistical analysis, age was dichotomized at the median. Differences in relative frequency of detected circulating colonic cells and correlation between qualitative variables were evaluated by means of the $\chi^2$ test, applying the Yates' correction when needed. Continuous variables with non-parametric distribution were compared by means of the Mann–Whitney *U*-test.

### RESULTS

### Recovery experiments

Tumour cells from a surgically resected colon carcinoma specimen were detected down to 5 cells per 10 ml blood by RT-PCR for CEA mRNA. This indicates a sensitivity limit of approximately one tumour cell per $10^7$ white blood cells. There was an increase in band intensity with increasing amounts of RNA, and no transcripts were identified in the negative control sample (Figure 2).

### CEA mRNA from clinical samples

CEA mRNA was detected in the peripheral blood samples from 39 out of 95 colorectal cancer patients (41.1%), probably indicating the presence of circulating neoplastic cells. According to the TNM classification, the frequency of positive cases was: two out of six patients (33%) in stage I, 12 of 32 patients (37.5%) in stage II, 13 of 37 patients (35.1%) in stage III, and 12 of 20 patients (60%) in stage IV. Correlation between CEA mRNA expression in peripheral blood and baseline characteristics of patients with colorectal cancer is depicted in Table 1. As shown, CEA mRNA positivity significantly correlated with the presence of distant metastases at inclusion [presence: 12/20 (60%) vs absence: 27/75 (36%); $P = 0.05$]. In addition, serum CEA levels in patients with CEA mRNA positivity (73 ± 283 ng $ml^{-1}$) were significantly higher than in those patients with negative expression (38 ± 179 ng $ml^{-1}$) (*U*-value: 476; $P < 0.02$).

In contrast, none of the healthy volunteers and only 1 of 11 patients (9.1%) with other gastrointestinal neoplasms had detectable CEA mRNA in peripheral blood. By contrast, CEA mRNA was detected in five of the nine patients (55.6%) with inflammatory bowel disease (Figure 3). These positive control subjects included one patient with gastric cancer, one patient with ulcerative colitis and four patients with Crohn's disease respectively.

Finally, in a non-selected subgroup of 24 CEA mRNA-positive patients submitted to surgical resection of the primary tumour, persistence of CEA mRNA expression was observed in 15 cases within 24 h after the intervention. Moreover, the prevalence of persistent CEA mRNA positivity was higher in patients with lymph node involvement and/or distant metastasis at surgery than

**Figure 2** Detection of CEA mRNA in 10 ml of normal blood sample spiked with dilutions of isolated colorectal cancer cells (recovery experiment). Electrophoresis on 2% agarose gel, followed by ethidium bromide staining. There was an increase in the intensity of the 388-bp band with increasing mRNA concentration. WM, molecular weight marker V; C+, positive control; C–, negative control

**Figure 3** Detection of CEA mRNA in peripheral blood of colorectal cancer patients and control subjects. Electrophoresis on 2% agarose gel, followed by ethidium bromide staining. RT-PCR products for GAPDH of the same RNA samples were identified as a 169-bp band. (**A**) WM, molecular weight marker V; C+, positive control; 1–4, positive colorectal cancer patients; 5 and 6, negative colorectal cancer patients. (**B**) WM, molecular weight marker V; 7 and 8, positive control patients (inflammatory bowel disease); 9 and 10, negative control patients (gastric and pancreatic carcinomas); 11 and 12, negative control subjects (healthy volunteers); C–, negative control

*© Cancer Research Campaign 1998*

in those in whom neoplastic dissemination was ruled out [12/17 (71%) vs 3/7 (43%) respectively; $P = 0.1$].

All cases demonstrated satisfactory RNA quality by a control PCR amplification of GADPH mRNA (Figure 3). No inconsistent results were obtained when repeating the analysis in the same sample.

### DISCUSSION

The results of the present study confirm that CEA mRNA can be detected in the peripheral blood of patients with colorectal carcinoma by means of RT-PCR. In this setting, the detection of CEA mRNA is very probably associated with the presence of circulating tumour cells on the basis of the following considerations: (1) the frequency of CEA mRNA positivity correlated with tumour stage; (2) CEA mRNA expression disappeared after surgical resection of the primary tumour in most of the non-metastatic colorectal cancer patients; (3) serum CEA concentration correlated with CEA mRNA positivity; and (4) healthy volunteers had undetectable CEA mRNA in the blood. Additionally, the recovery experiment indicated a clear correlation between CEA mRNA expression and tumour cell concentration. In this regard, although it cannot be assumed that the detection threshold in all clinical samples was identical to that in the recovery assay (O'Sullivan et al, 1995), results obtained in colorectal cancer patients and healthy volunteers indicate that specificity of our approach seems to be adequate for its purpose. Nevertheless, false-negative results could not be definitively ruled out.

Several reports have demonstrated that PCR is a useful tool for the detection of circulating cancer cells in solid tumours (Johnson et al, 1995). In gastrointestinal tumours, in which cancer-specific mutations in DNA have not yet been found, it is necessary to use reverse transcriptase to prepare cDNA from peripheral blood mRNA for identifying tissue-specific gene expression. Detection of occult neoplastic cells in patients with colorectal cancer has been carried out using different targets. K-*ras* mutations have been reported as a useful marker when samples of the primary tumour are available to confirm the mutation (Hardingham et al, 1995). Cytokeratins, a multigene family of proteins with differentiation-associated patterns of expression (Moll et al, 1982), have also been used to characterize neoplastic cells of epithelial origin in bone marrow (Lindemann et al, 1992) and peripheral blood (Denis et al, 1997; Nakamori et al, 1997; Soeth et al, 1997). However, most of them (CK-8, CK-18 and CK-19) are found in samples of healthy subjects, thus limiting their suitability as targets (Burchill et al, 1995). CEA was chosen as a target because it is a cell-surface molecule constitutively expressed in the colonic epithelial tissue, and its expression is maintained in almost all colorectal carcinomas (Shively and Beatty, 1985). Its genomic organization (Schrewe et al, 1990) and cDNA sequence (Oikawa et al, 1987) are known. In addition, this target has been used in the specific detection of CEA-expressing tumour cells in bone marrow aspirates (Gerhard et al, 1994) and, recently, in the identification of circulating neoplastic cells in patients with colorectal liver metastasis (Jonas et al, 1996).

From a clinical point of view, the present study documents limitations on both the sensitivity as well as the specificity of the assay, thus probably restricting the diagnostic usefulness of this technique in colorectal cancer. In that sense, CEA mRNA could be detected in patients with other gastrointestinal neoplasms expressing CEA as it was expected, and in those with inflammatory bowel disease. By contrast, not all colorectal cancer patients, including those with stage IV disease, had detectable CEA mRNA expression. This fact probably reflects that tumour cells are circulating in clusters (Liotta and Stetler-Stevenson, 1991), thus favouring a bias in the collection of blood samples. According to this suggestion, the true prevalence of circulating cancer cells is likely to be much higher than that detected by a single blood extraction.

In contrast, the presence of circulating tumour cells is expected to be associated with a poor prognosis (Hardingham et al, 1995), in a similar manner to micrometastatic tumour cells in bone marrow (Lindemann et al, 1992; Soeth et al, 1997). Results of the present investigation suggest that circulating neoplastic cells correlate with the presence of distant metastasis at inclusion. However, it should be emphasized that a high proportion of non-metastatic colorectal cancer patients (stages I–III) were positive for CEA mRNA expression, even after surgery. Then, clinical relevance of this finding remains uncertain at present. Considering metastasis is believed to be an inefficient process (Liotta and Stetler-Stevenson, 1991), evidence of circulating tumour cells could represent an irrelevant clinical event. On the contrary, if their presence had prognostic implications, detection of malignant cells in peripheral blood may improve tumour staging and help to identify those patients who would benefit from systemic therapy after surgery. Accordingly, the presence of circulating cancer cells should be considered either as a marker of metastatic potential or evidence of residual disease, and redefinition of remission status could be needed. Long-term follow-up of these patients will allow us to establish its clinical relevance.

In the present study, a significant proportion of patients with inflammatory bowel disease had detectable CEA mRNA expression in the blood, the significance of this finding being unclear. Illegitimate transcription by white blood cells or epithelial contamination of samples with a small number of skin cells because of the venepuncture cannot be formally excluded, but the absence of expression in healthy subjects made this possibility very unlikely. Circulating CEA mRNA caused by cytolysis is improbable because of large amounts of RNAases (Hillaire et al, 1994). Although up-regulation of CEA gene family members in granulocytes of patients with inflammatory bowel disease has not been described yet, the feasibility of this hypothesis cannot be ruled out. However, considering that this disease involves necrosis of the colonic epithelium, CEA mRNA expression suggests the presence of non-neoplastic epithelial colonic cells in peripheral blood. A similar event has been shown among patients with non-malignant disease submitted to surgical resection or liver transplantation in whom peripheral alpha-fetoprotein mRNA expression was detected after surgery (Lemoine et al, 1997). Unfortunately, the low sensivity of both direct microscopy and immunocytochemistry in this setting (Leather et al, 1993; Wong et al, 1995) precludes the distinction of both cellular subtypes and, therefore, a definitive assessment of the origin of CEA mRNA expression in patients with inflammatory bowel disease.

In summary, the results of the present investigation confirm that it is possible to amplify CEA mRNA in the peripheral blood, its presence in colorectal cancer patients being almost certainly derived from circulating neoplastic cells. Correlation of this cellular subpopulation with the occurrence of distant metastasis at inclusion suggests a possible role of this method in improving cancer staging. Nevertheless, CEA mRNA detectable in blood of patients with inflammatory bowel disease probably indicates the presence of non-neoplastic epithelial colonic cells. Therefore,

© Cancer Research Campaign 1998

CEA mRNA expression would indicate the loss of cell adhesion in colonic epithelium because of either neoplastic or inflammatory processes.

### ACKNOWLEDGEMENTS

This work was supported in part by grants from the Fondo de Investigaciones Sanitarias de la Seguridad Social (96/0240) and the Plan Nacional de Investigación Científica y Desarrollo Tecnológico (SAF97-0107). Loreto Boix and Laura Gargallo received a research grant from the Marató TV3-Càncer (95/3008).

### REFERENCES

Ballet F. Bouma ME. Wang SR. Amit N. Marais J and Infante R (1984) Isolation. culture and characterisation of adult human hepatocytes from surgical liver biopsies. *Hepatology* **4**: 849–854

Boring CC. Squires TS and Tong T (1993) Cancer statistics. 1993. *CA Cancer J Clin* **43**: 7–26

Burchill SA. Bradbury MF. Pittman K. Southgate J. Smith B and Selby P (1995) Detection of epithelial cancer cells in peripheral blood by reverse transcriptase polymerase chain reaction. *Br J Cancer* **71**: 278–281

Chirgwin JM. Przybyla AE. McDonald RJ and Rutter WJ (1979) Isolation of biologically active ribonucleic acid from sources enriched in ribonuclease. *Biochemistry* **18**: 5294–5299

Denis MG. Lipart C. Leborgne J. LeHur PA. Galmiche JP. Denis M. Ruud E. Truchaud A and Lustenberger P (1997) Detection of disseminated tumor cells in peripheral blood of colorectal cancer patients. *Int J Cancer* **74**: 540–544

Gerhard M. Juhl H. Kalthoff H. Schreiber HW. Wagener C and Neumaier M (1994) Specific detection of carcinoembryonic antigen-expressing tumor cells in bone marrow aspirates by polymerase chain reaction. *J Clin Oncol* **12**: 725–729

Hardingham JE. Kotasek D. Sage RE. Eaton MC. Pascoe VH and Dobrovic A (1995) Detection of circulating tumor cells in colorectal cancer by immunobead-PCR is a sensitive prognostic marker for relapse of disease. *Mol Med* **1**: 789–794

Hillaire S. Barbu V. Boucher E. Moukhtar M and Poupon R (1994) Albumin messenger RNA as a marker of circulating hepatocytes in hepatocellular carcinoma. *Gastroenterology* **106**: 239–242

Johnson PWM. Burchill SA and Selby PJ (1995) The molecular detection of circulating tumour cells. *Br J Cancer* **72**: 268–276

Jonas S. Windeatt S. O-Boateng A. Fordy C and Allen-Mersh TG (1996) Identification of carcinoembryonic antigen-producing cells circulating in the blood of patients with colorectal carcinoma by reverse transcriptase polymerase chain reaction. *Gut* **39**: 717–721

Leather AJM. Gallegos NC. Kocjan G. Savage F. Smales CS. Hu W. Boulos PB. Northover JMA and Phillips RKS (1993) Detection and enumeration of circulating tumour cells in colorectal cancer. *Br J Surg* **80**: 777–780

Lemoine A. Le Bricon T. Salvucci M. Azoulay D. Pham P. Raccuia J. Bismuth H and Debuire B (1997) Prospective evaluation of circulating hepatocytes by alpha-fetoprotein mRNA in humans during liver surgery. *Ann Surg* **226**: 43–50

Lindemann F. Schlimok G. Dirschedl P. Witte J and Riethmüller G (1992) Prognostic significance of micrometastatic tumour cells in bone marrow of colorectal cancer patients. *Lancet* **340**: 685–689

Liotta LA and Stetler-Stevenson WG (1991) Tumor invasion and metastasis: an imbalance of positive and negative regulation. *Cancer Res* **51**: 5054s–5059s

Moll R. Franke WW and Schiller DL (1982) The catalog of human cytokeratins: patterns of expression in normal epithelia. tumors and cultured cells. *Cell* **31**: 11–24

Moreno JG. Croce CM. Fischer R. Monne M. Vihko P. Mulholland SG and Gomella LG (1992) Detection of hematogenous micrometastasis in patients with prostate cancer. *Cancer Res* **52**: 6110–6112

Nakamori S. Kameyama M. Furukawa H. Takeda O. Sugai S. Imaoka S and Nakamura Y (1997) Genetic detection of colorectal cancer cells in circulation and lymph nodes. *Dis Colon Rectum* **40**: S29–S36

Oikawa S. Nakazato H and Kosaki G (1987) Primary structure of human carcinoembryonic antigen deduced from cDNA sequence. *Biochem Biophys Res Commun* **142**: 511–518

O'Sullivan GC. Collins JK. O'Brien F. Crowley B. Murphy K. Lee G and Shanahan F (1995) Micrometastases in bone marrow of patients undergoing 'curative' surgery for gastrointestinal cancer. *Gastroenterology* **109**: 1535–1540

Roberts E. Letarte M. Squire J and Yang S (1994) Characterization of human hepatocyte lines derived from normal liver tissue. *Hepatology* **19**: 1390–1399

Safi F and Beyer HG (1993) The value of follow-up of curative surgery of colorectal carcinoma. *Cancer Detect Prev* **17**: 417–424

Schrewe H. Thompson J. Bona M. Hefta LJF. Maruya A. Hassauer M. Shively JE. Von Kleist S and Zimmermann W (1990) Cloning of the complete gene for carcinoembryonic antigen: analysis of its promoter indicates a region conveying cell type-specific expression. *Mol Cell Biol* **10**: 2738–2748

Shively JE and Beatty JD (1985) CEA-related antigens: molecular biology and clinical significance. *Crit Rev Oncol Hematol* **2**: 355–399

Smith B. Selby P. Southgate J. Pittman K. Bradley C and Blair GE (1991) Detection of melanoma cells in peripheral blood by means of reverse transcriptase and polymerase chain reaction. *Lancet* **338**: 1227–1229

Soeth E. Vogel I. Roder C. Juhl H. Marxsen J. Kruger U. Henne-Bruns D. Kremer B and Kalthoff H (1997) Comparative analysis of bone marrow and venous blood isolates from gastrointestinal cancer patients for the detection of disseminated tumor cells using reverse transcription PCR. *Cancer Res* **57**: 3106–3110

Tada M. Omata M. Kawai S. Saisho H. Ohto M. Saiki RK and Sninsky JJ (1993) Detection of *ras* gene mutations in pancreatic juice and peripheral blood of patients with pancreatic adenocarcinoma. *Cancer Res* **53**: 2472–2474

Wong LS. Bateman WJ. Morris AG and Fraser IA (1995) Detection of circulating tumour cells with the magnetic activated cell sorter. *Br J Surg* **82**: 1333–1337

Wong LS. Cantrill JE. Odogwu S. Morris AG and Fraser IA (1997) Detection of circulating tumour cells and nodal metastasis by reverse transcriptase polymerase chain reaction technique. *Br J Surg* **84**: 834–839

© Cancer Research Campaign 1998